# Using verbal autopsy to measure causes of death: the comparative performance of existing methods

**DOI:** 10.1186/1741-7015-12-5

**Published:** 2014-01-09

**Authors:** Christopher JL Murray, Rafael Lozano, Abraham D Flaxman, Peter Serina, David Phillips, Andrea Stewart, Spencer L James, Alireza Vahdatpour, Charles Atkinson, Michael K Freeman, Summer Lockett Ohno, Robert Black, Said Mohammed Ali, Abdullah H Baqui, Lalit Dandona, Emily Dantzer, Gary L Darmstadt, Vinita Das, Usha Dhingra, Arup Dutta, Wafaie Fawzi, Sara Gómez, Bernardo Hernández, Rohina Joshi, Henry D Kalter, Aarti Kumar, Vishwajeet Kumar, Marilla Lucero, Saurabh Mehta, Bruce Neal, Devarsetty Praveen, Zul Premji, Dolores Ramírez-Villalobos, Hazel Remolador, Ian Riley, Minerva Romero, Mwanaidi Said, Diozele Sanvictores, Sunil Sazawal, Veronica Tallo, Alan D Lopez

**Affiliations:** 1 Institute for Health Metrics and Evaluation, University of Washington, 2301 5th Avenue Suite 600, Seattle, WA 98121, USA; 2 National Institute of Public Health, Universidad 655, 62100 Cuernavaca, Morelos, Mexico; 3 Johns Hopkins University, Bloomberg School of Public Health, 615 N Wolfe St #5041, Baltimore, MD 21205, USA; 4 Public Health Laboratory-IdC, P.O. BOX 122 Wawi Chake Chake Pemba, Zanzibar, Tanzania; 5 Public Health Foundation of India, ISID Campus, 4 Institutional Area, Vasant Kunj, New Delhi 110070, India; 6 Brigham and Women's Hospital, 75 Francis St, Boston, MA 02215, USA; 7 Global Development, Bill and Melinda Gates Foundation, PO Box 23350, Seattle, WA 98012, USA; 8 CSM Medical University, Shah Mina Road, Chowk, Lucknow, Uttar Pradesh 226003, India; 9 Dept of International Health, Johns Hopkins Bloomberg School of Public Health, E5521, 615 N. Wolfe Street, Baltimore, MD 21205, USA; 10 Public Health Laboratory-Ivo de Carneri, Wawi, Chake-Chake, Pemba, Zanzibar, Tanzania; 11 Johns Hopkins University, 214A Basement, Vinobapuri Lajpat Nagar-II, New Delhi 110024, India; 12 Harvard School of Public Health, 677 Huntington Avenue, Boston, MA 02115-6018, USA; 13 The George Institute for Global Health, The University of Sydney, 83/117 Missenden Rd, Camperdown, NSW 2050, Australia; 14 Community Empowerment Lab, Shivgarh, India; 15 Research Institute for Tropical Medicine, Corporate Ave, Muntinlupa City 1781, Philippines; 16 Division of Nutritional Sciences, Cornell University, 314 Savage Hall, Ithaca, NY 14853, USA; 17 The George Institute for Global Health, 839C, Road No. 44A, Jubilee Hills, Hyderabad 500033, India; 18 Muhimbili University of Health and Allied Sciences, United Nations Rd, Dar es Salaam, Tanzania; 19 School of Population Health, University of Queensland, Level 2 Public Health Building School of Population Health, Herston Road, Herston, QLD 4006, Australia; 20 University of Melbourne School of Population and Global Health, Building 379, 207 Bouverie St., Parkville 3010, VIC, Australia

**Keywords:** Verbal autopsy, VA, Validation, Cause of death, Symptom pattern, Random forests, InterVA, King-Lu, Tariff

## Abstract

**Background:**

Monitoring progress with disease and injury reduction in many populations will require widespread use of verbal autopsy (VA). Multiple methods have been developed for assigning cause of death from a VA but their application is restricted by uncertainty about their reliability.

**Methods:**

We investigated the validity of five automated VA methods for assigning cause of death: InterVA-4, Random Forest (RF), Simplified Symptom Pattern (SSP), Tariff method (Tariff), and King-Lu (KL), in addition to physician review of VA forms (PCVA), based on 12,535 cases from diverse populations for which the true cause of death had been reliably established. For adults, children, neonates and stillbirths, performance was assessed separately for individuals using sensitivity, specificity, Kappa, and chance-corrected concordance (CCC) and for populations using cause specific mortality fraction (CSMF) accuracy, with and without additional diagnostic information from prior contact with health services. A total of 500 train-test splits were used to ensure that results are robust to variation in the underlying cause of death distribution.

**Results:**

Three automated diagnostic methods, Tariff, SSP, and RF, but not InterVA-4, performed better than physician review in all age groups, study sites, and for the majority of causes of death studied. For adults, CSMF accuracy ranged from 0.764 to 0.770, compared with 0.680 for PCVA and 0.625 for InterVA; CCC varied from 49.2% to 54.1%, compared with 42.2% for PCVA, and 23.8% for InterVA. For children, CSMF accuracy was 0.783 for Tariff, 0.678 for PCVA, and 0.520 for InterVA; CCC was 52.5% for Tariff, 44.5% for PCVA, and 30.3% for InterVA. For neonates, CSMF accuracy was 0.817 for Tariff, 0.719 for PCVA, and 0.629 for InterVA; CCC varied from 47.3% to 50.3% for the three automated methods, 29.3% for PCVA, and 19.4% for InterVA. The method with the highest sensitivity for a specific cause varied by cause.

**Conclusions:**

Physician review of verbal autopsy questionnaires is less accurate than automated methods in determining both individual and population causes of death. Overall, Tariff performs as well or better than other methods and should be widely applied in routine mortality surveillance systems with poor cause of death certification practices.

## Background

Reliable information on the number of deaths by age, sex and cause is the cornerstone of an effective health information system [1,2]. Levels and trends in cause-specific mortality provide critical insights into emerging or neglected health problems and the effectiveness of current disease control priorities. Further, monitoring progress with national health development goals and global poverty reduction strategies enshrined in the Millennium Development Goals requires a reliable understanding of how leading causes of death are changing in populations. The urgency of supporting countries to implement reliable and cheap cause of death measurement strategies is becoming increasingly evident with the strong country leadership expectations that are driving the post-2015 development agenda. Yet with the remarkably slow progress over the last 40 years or so in the development of vital registration systems built on medical certification of causes of death, countries will be ‘driving blind’ [3]. The recent Report of the High-Level Panel on the post-2015 Development Agenda has called for a ‘data revolution’ [4] to urgently improve the quality and availability of information on key development indicators, including patterns of disease in populations, and to exploit new measurement and data collection technologies. Civil registration systems which are able to generate reliable vital statistics on the health of populations are central to the new emphasis on accountability, but there is little prospect of countries being able to do so if they continue to pursue current cause of death measurement strategies based on incrementally expanding coverage of physician certification of deaths.

How then, might countries accelerate cause of death measurement in their populations in order to monitor progress with their development goals and deliver on the promise of the ‘data revolution’ that is being called for? What is required are cheap, effective methods to reliably assess cause of death patterns that facilitate comparisons over time and with the evaluation of disease control strategies. Moreover, these methods need to be capable of realistic application in the poorest populations where physician availability is likely to be extremely limited, thus ensuring compliance with a key tenant of the post-2015 development strategy to ‘leave no one behind’ [4].

A death certificate completed by a physician with substantial knowledge of the clinical course of an individual prior to death based on appropriate diagnostics is the *de facto* standard for cause of death assignment. When deaths occur outside of a hospital or occur in facilities with limited diagnostic capability, verbal autopsy (VA) has increasingly been proposed and used to measure cause of death patterns. Recent studies suggest that VA can provide cause of death information that, at the population level, is similar to death certification in high-quality hospitals [5]. VA is thus a potential data collection option for low-resource settings to confidently monitor progress with their development strategies, provided it can be shown to be realistic, reliable and routinely applicable.

Interest in VA as a tool for monitoring causes of death in research settings has grown steadily. For example, the number of articles referring to VA in Google Books has doubled every five-year period over the last two decades [6]. More recently, several developing country governments, including India, Brazil, and Sri Lanka, have used forms of VA in official data collection systems. Mozambique has implemented a national VA sample as part of their decennial census [7]. Other countries such as Zambia and Tanzania are developing national sample registration systems using VA, and China has already done so [8,9]. The World Health Organization (WHO) has called for wider use of VA specifically to track the non-communicable disease epidemic in many developing countries without adequate death registration and medical certification [10]. The increased use of VA for routine application in national health information systems has the potential to greatly improve the availability of reliable and essential information on causes of death for disease control programs worldwide but has been constrained by widespread concerns about the dependability of symptom information collected from families and the practicality of relying on physicians to review anonymous symptom-based questionnaires. Confidence in VA as a legitimate data collection mechanism has been limited because it is not known how accurately the method can diagnose the underlying cause of death compared with hospital-based procedures or how different approaches to VA perform in assigning causes of death.

VA encompasses a diverse set of tools. An instrument is used to conduct the interview of family members about their recollection of signs, symptoms and characteristics of the individual and events prior to death, as well as the decedent’s experience of health care. Then, an analytical method is used to process the information collected in the interview in order to diagnose the cause of death. WHO has recently proposed a standardized instrument, [11] variants of which have been used in a number of demographic surveillance sites [12] and in the Population Health Metrics Research Consortium (PHMRC) VA validation study, which collected more than 12,535 VA interviews for deaths where the true underlying cause was reliably known through pre-defined rigorous diagnostic criteria [13]. The validity of at least six analytical methods to assign cause has been studied using comparable data from the PHMRC study: physician-certified VA (PCVA), InterVA 3.2, King-Lu (KL) direct cause-specific mortality fraction (CSMF) estimation, the Tariff method (Tariff), Random Forest (RF), and the Simplified Symptom Pattern (SSP) method [14-19].

PCVA is the traditional approach to verbal autopsy and uses the judgment of a physician to determine the most likely cause of death based on a verbal autopsy. InterVA is an application of Bayes’ Theorem that uses expert review panels to determine the probability of saying yes to each item conditional on the true cause of death. The King-Lu method uses information on the probability of saying yes to each item from a reference dataset to estimate the cause fractions in a population sample but does not assign cause at the individual level. The Tariff method calculates a score, or tariff, for each symptom-cause pair based on observed endorsement rates in the data that effectively identify the symptoms with a strong ‘signal’ for each cause. Random Forest uses a machine learning algorithm to classify causes of death based on the automated creation of decision trees. Simplified Symptom Pattern is a statistical implementation of Bayes’ Theorem that takes into account symptom clustering. Performance of all methods was assessed using new metrics [20] and a broad set of test datasets that are meant to generate more robust assessments across a range of cause of death compositions.

PCVA is the current practice in most VA applications, but it is expensive and inefficient to apply since it relies on physician review of VA forms. However, until now, PCVA has been considered the method of choice if resources allow. In this paper, we take advantage of the recent series of method-specific studies that have been published and the PHMRC validation dataset, to investigate the comparative performance of available VA methods, including PCVA [14-19]. We use any revisions of these methods, such as InterVA-4, [21] that have emerged since the original PHMRC publications to provide an objective, comprehensive and up-to-date comparison of the performance of various methods in diagnosing VAs. This comparative information on performance and the relative strengths and weaknesses of various methods is intended to facilitate choices by researchers and managers of health information systems wishing to deploy VA as a tool for routinely monitoring causes of death in their populations.

## Methods

The design, implementation, and broad findings from the PHMRC Gold Standard Verbal Autopsy validation study are described elsewhere [13]. Briefly, the study collected VAs in six sites: Andhra Pradesh and Uttar Pradesh in India, Bohol in the Philippines, Mexico City in Mexico, and Dar es Salaam and Pemba Island in Tanzania. Gold standard (GS) clinical diagnostic criteria were specified by a committee of physicians for 53 adult, 27 child and 13 neonatal causes plus stillbirths prior to data collection. Deaths fulfilling the GS criteria were identified in each of the sites. It is important to note that the stringent diagnostic criteria used in this validation study differ from traditional validation studies, which frequently use physician judgment to certify deaths based on available clinical records. Even if independent clinicians are used to certify the cause of death, the diagnosis is subjective in nature, non-standardized and further limited by any biases of the individual clinician and the availability of diagnostic tests. Once the GS deaths that met the criteria were identified, VA interviews were then conducted with household members by interviewers who had no knowledge of the cause of death. Separate modules were used for adults, children and neonates [13]. The PHMRC instrument was based on the WHO recommended VA instrument with some limited modifications [13].

At the end of the study, 12,535 verbal autopsies on deaths with GS diagnoses were collected (7,846 adults, 2,064 children, 1,620 neonates and 1,005 stillbirths). This is seven fewer than previously published due to final revision of the preliminary dataset. Additional revisions include recoding several items in the dataset including the question ‘Did decedent suffer from an injury?’ which was considered an endorsement conditional on the injury occurring within thirty days of death. Questions not directly related to cause of death, such as ‘Was care sought outside the home?’, are no longer used in order to avoid potential bias when analyzing data sets from other populations.

Additional files 1, 2 and 3: Tables S1a to S1c provide information on the number of GS deaths collected for adults, children and neonates by cause and by diagnostic level. The study protocol defined three levels of cause of death assignment based on the diagnostic documentation: Level 1, 2A and 2B. Level 1 diagnoses are the highest level of diagnostic certainty possible for that condition, consisting of either an appropriate laboratory test or X-ray with positive findings, as well as medically observed and documented illness signs. Level 2A diagnoses are of moderate certainty, consisting of medically observed and documented illness signs. Level 2B was used rarely in place of level 2A if medically observed and documented illness signs were not available but records nonetheless existed for treatment of a particular condition. Details of the clinical and diagnostic criteria for each cause have been published [13]. Of all GS deaths collected, 88% met Level 1 criteria, which we used for all primary analysis. In various sensitivity analyses that have been conducted, the results do not differ when only Level 1 deaths are used compared to all deaths. Because of small numbers of deaths collected for some causes, we were able to estimate causes of death and evaluate the methods for 34 causes for adults, 21 causes for children and 5 causes for neonates plus stillbirths [13]. The choice of the causes used in the study is elaborated elsewhere [13]. The number of neonatal causes evaluated was reduced from 10 to 5, excluding stillbirths, because of the use of combinations of causes that do not map to the International Classification of Diseases and Injuries (ICD). Results from these analyses are presented based on the Global Burden of Disease (GBD) 2010 cause list, which divides causes of death into three broad groups: communicable, maternal, neonatal and nutritional disorders; non-communicable diseases; and injuries [22].

The VA data, consisting of both the interview and open narrative, were sent to physicians at each data collection site who were trained to fill out standardized death certificates for each VA interview. Substantial efforts were taken to standardize PCVA across sites including using standardized training material and the same trainers. Further details on these efforts to standardize PCVA are described in detail elsewhere [14]. In addition to the standard VA, we sent VAs excluding the open narrative and information on the recall of health care experience to a different set of physicians to test what would be the performance of PCVA in settings where decedents had had limited contact with health services.

**Figure 1:**
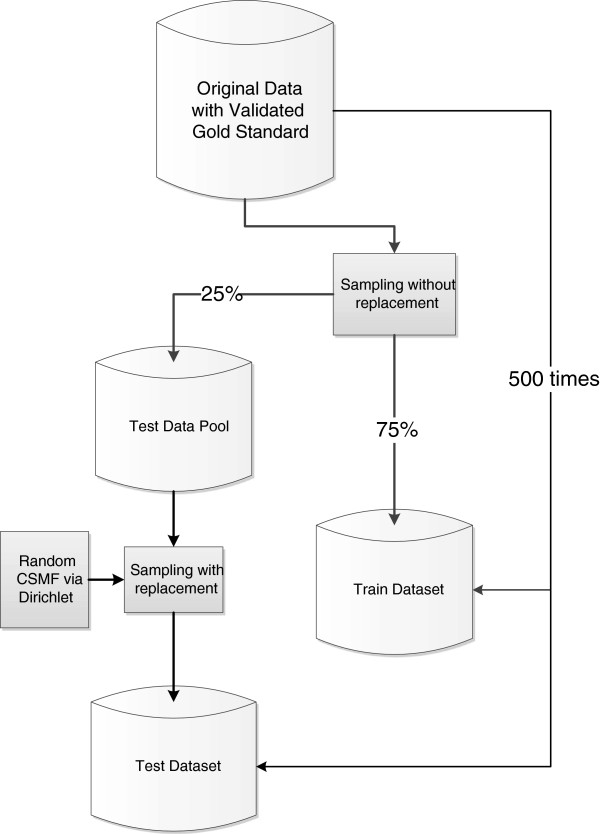
**Process of generating 500 test and train validation datasets.** A detailed flowchart illustrating the process by which 500 different populations with different cause distributions were simulated in order to validate the analytical models in 500 separate scenarios.

A well-known problem with the analysis of VAs is that performance may vary as a function of the true cause of death composition in the population studied. To avoid this limitation, as part of the PHMRC study, 500 train-test data analysis datasets were generated. Each train-test pair has a different true cause of death composition. Figure 1 illustrates how the validation data have been used to generate each train-test pair. This procedure ensures that in each train-test dataset pair, the train set and test set contain no deaths in common. It further guarantees that there is no correlation between the CSMF composition in the train set and the test set. This is important because some automated methods can yield exaggerated performance when the test and train datasets have similar cause compositions [20,23].

As noted, the process of separating the data into test and train datasets was repeated 500 times to eliminate the influence of cause composition on the results of our analysis. Each of the 500 test data sets has a different cause composition and analysis of all 500 datasets results in a distribution of the metrics of performance, from which we can calculate overall metrics and their uncertainty intervals. By analyzing performance of methods across multiple pairs of train-test datasets, we can ensure that conclusions about comparative performance are not biased by the particular cause composition of the test dataset. All methods except InterVA-4 have been compared using exactly the same train-test datasets, and all methods except InterVA-4 have been compared using exactly the same cause lists. InterVA-4 yields cause assignments for a different list of causes than the list developed for the PHMRC study [21].

Since the publication of the study on the comparative performance of InterVA 3.2 [15], InterVA-4 [21] has been released. InterVA-4 includes a longer list of possible cause assignments than InterVA 3.2, including maternal and stillbirth causes. In this study, we use InterVA-4 for comparison. The cause list has changed slightly between InterVA 3.2 and InterVA-4. Therefore, the mapping of the PHMRC cause list to the InterVA-4 cause list has also been revised. This new cause mapping is described in Additional files 4, 5 and 6: Tables S2a to S2c. The new cause mapping requires a ‘joint cause list,’ which is a shorter list than the PHMRC cause list. When shorter lists are used, a method will usually perform better than when longer lists are used so performance for InterVA-4 may be exaggerated.

The Tariff method has also been updated so that only tariffs that are statistically significant are used to generate a tariff score for a death. This revision along with other slight modifications is explained in detail in Additional file 7. RF and SSP use tariff scores as an input into their algorithms so the revisions to Tariff slightly modify the performance of these automated methods as well.

Performance of each method has been assessed in two dimensions: how well methods assign cause of death correctly for individual deaths and how accurately CSMFs are estimated for populations. For assessing the performance of each method at assigning true cause of death for individual deaths for specific causes, we report sensitivity and specificity. Because these measures, particularly specificity, are a function of the underlying cause of death structure in the population, we report the median value of each metric and provide in additional tables further detail across the 500 splits. To summarize performance of each method at assigning deaths to the correct cause across all causes, we report two overall measures: chance-corrected concordance (CCC) and Cohen’s Kappa [20]. Both summary measures count how often a cause is correctly assigned and then adjust for how often this is expected on the basis of chance. Chance-corrected concordance for cause j (CCC_j_) is measured as:

$$\mathrm{CC}C_{j}=\frac{\left(\frac{TP_{j}}{TP_{j}+FN_{j}}\right)-\left(\frac{1}{N}\right)}{1-\left(\frac{1}{N}\right)}$$

where TP is true positives, FN is false negatives, and N is the number of causes. TP plus FN equals the true number of deaths from cause j.

**Figure 2:**
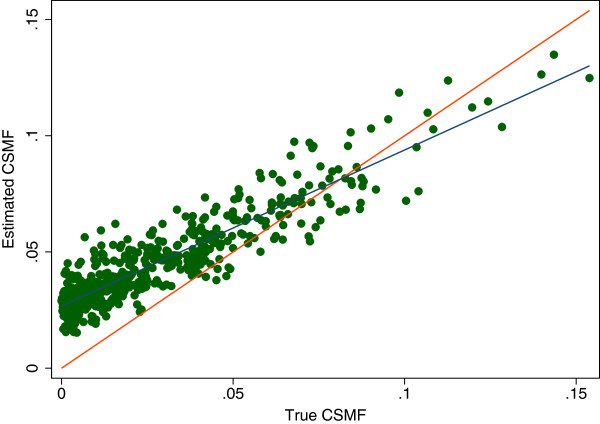
**Estimated cause-specific mortality fraction (CSMF) versus true CSMF example.** A graphical example of the regression of the estimated over true CSMF. This particular example is for the estimation of epilepsy using SSP without HCE. Each dot represents a single split, or simulated population, and SSP’s estimate of fraction of epilepsy in the population as compared to the true fraction. The red line represents a perfect estimate while the blue line represents a line of best fit for the data. HCE, health care experience; SSP, Simplified Symptom Pattern.

For many purposes, it is more important to assess how well a VA method does in estimating CSMFs. For individual causes, we compare the true CSMF and the estimated CSMF by regressing estimated CSMF on true CSMF and report the slope, intercept and root mean square error (RMSE) of this regression. If a method perfectly predicts the true CSMF, the slope would be 1.0, the intercept would be zero and RMSE would be zero. This concept is illustrated in Figure 2 which shows a comparison of estimated CSMFs and true CSMF for one cause for one method. Each point represents the true and estimated CSMF for a cause from one of the 500 splits. The red line represents the circumstances where the true CSMF would equal the estimated CSMF. The blue line is the linear regression line fit to the observed relationship between the true and estimated CSMFs. As indicated, the slope will tend to be less than one when sensitivity is reduced; likewise, the intercept will tend to be non-zero when specificity is reduced. The exact impact, however, is also a function of the correlation structure of misclassification errors.

To summarize overall performance of a method across causes, we compute the CSMF accuracy for each of the 500 test datasets, defined as [20]:

$$\mathrm{CSMF}\;\mathrm{Accuracy}=1-\frac{\sum_{j=1}^{k}\left|\mathrm{CSM}F_{j}^{\mathrm{true}}\right)-\left(\mathrm{CSM}F_{j}^{\mathrm{prea}}\right|}{2\left(1-\mathrm{Minimum}\;\left(\mathrm{CSM}F_{j}^{\mathrm{true}}\right)\right)}$$

As defined, CSMF accuracy will be 1 when the CSMF for every cause is predicted with no error. CSMF accuracy will be zero, when the summed errors across causes reach the maximum possible. To summarize overall performance of a method in predicting CSMFs that is robust to variation in the cause composition in the population, we report the median CSMF accuracy across the 500 splits.

Performance was also assessed with and without household recall of health care experience (HCE), if any, prior to death. HCE includes information about the cause of death or other characteristics of the illness told to the family by health care professionals transmitted in the open section of the instrument or evidence from medical records retained by the family and the responses to questions specifically related to disease history including all questions from the section 1 of the Adult module, such as ‘Did the deceased have any of the following: Cancer’ [13]. The open text information was parsed and tokenized using the Text Mining Package in R version 2.14.0 [24]. The resulting information is a series of dichotomous variables indicating that a certain word was included in the open text. By excluding from the analysis information on the household experience of health care, the applicability of various methods in populations with limited or no access to care may be approximated. However, it is possible that the process of contact with health services may also change responses to other items in the instrument.

Performance of methods varies depending on the underlying CSMF composition in the test population. In other words, for a given CSMF composition one method may outperform another even if in most cases the reverse is true. To quantify this, we assess which method performs best for CCC and CSMF accuracy for each of the 500 test data sets (which have different cause compositions). We also compute which method has the smallest absolute CSMF error for each cause across the 500 splits. This provides an evaluation of how often the assessment of which method works best is a function of the true CSMF composition of the test data and which method performs best for a specific cause.

## Results

### Adults

**Figure 3:**
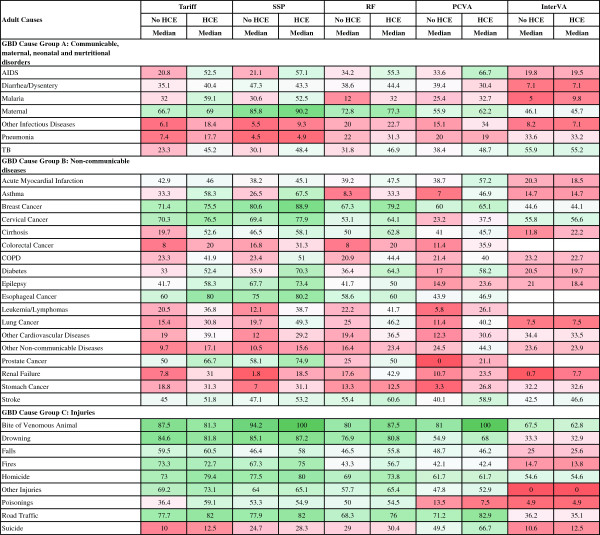
**Sensitivity (%) for 5 methods for 34 adult causes.** Figure 3 shows the median sensitivity for each method (except King-Lu which does not provide individual cause of death assignments) for 34 adult causes. Cells are shaded from red (low sensitivity) to green (high sensitivity) to help identify the relative differences between sensitivities across methods and causes. COPD, chronic obstructive pulmonary disease; GBD, Global Burden of Disease; HCE, health care experience; PCVA, physician certified VA; RF, Random Forest; SSP, Simplified Symptom Pattern; TB, tuberculosis.

**Figure 4:**
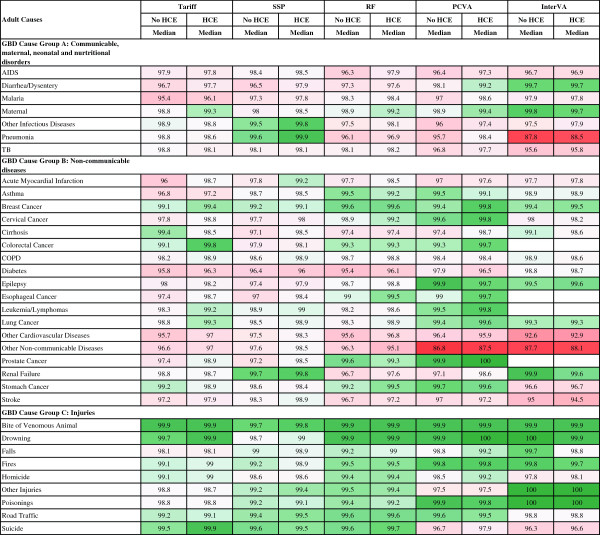
**Specificity (%) for 5 methods for 34 adult causes.** Figure 4 shows the median specificity for each method (except King-Lu which does not provide individual cause of death assignments) for 34 adult causes. Cells are shaded from red (low specificity) to green (high specificity) to help identify the relative differences between specificities across methods and causes. COPD, chronic obstructive pulmonary disease; GBD, Global Burden of Disease; HCE, health care experience; PCVA, physician-certified VA; RF, Random Forest; SSP, Simplified Symptom Pattern; TB, tuberculosis; VA, verbal autopsy.

Figure 3 reports the median sensitivity of each method (except King-Lu which does not provide individual cause of death assignments) for each cause and Figure 4 reports the median specificity of each method for each cause. Cells in these figures have been color coded to help identify patterns across methods and causes – green for higher values and red for lower values. Several general patterns emerge across methods and causes. InterVA-4 has much lower sensitivities than any of the other methods for nearly all causes. Among the other four methods, there is considerable correlation in sensitivities across the methods by cause, suggesting that it is easier, regardless of method, to assign some causes correctly. For example, sensitivities are relatively high for AIDS with HCE, maternal causes, breast cancer, esophageal cancers, and most of the injuries except for suicide. SSP yields very high sensitivities (over 70%) with the inclusion of HCE for five injuries; maternal causes; breast, cervical, esophageal and prostate cancers; diabetes; and epilepsy. Many other causes have sensitivities above 50%, while some causes, especially residual categories such as other non-communicable diseases, have quite low sensitivities. There is substantial variation in performance for specific causes across the methods as well. For example, for malaria, sensitivity ranges from 9.8% for InterVA-4 with HCE to 59.1% for Tariff with HCE. PCVA has the highest sensitivity for suicide and for the residual categories other non-communicable diseases and other infectious diseases.

Figure 4 indicates particular weaknesses for methods where specificity drops below 95% which will lead to substantial over-estimation of CSMFs for these causes: PCVA for other non-communicable, InterVA-4 for pneumonia, other cardiovascular and other non-communicable. Specificities in the 95% to 98% range are also problematic and these are noticeable for many causes including major public health challenges, such as malaria. Additional file 8: Table S3 provides the standard deviation of sensitivity and specificity by cause and method across the 500 splits indicating that both sensitivity and specificity can vary as a function of the cause composition of the population and due to stochastic variation in the deaths selected in the train and test splits.

**Figure 5:**
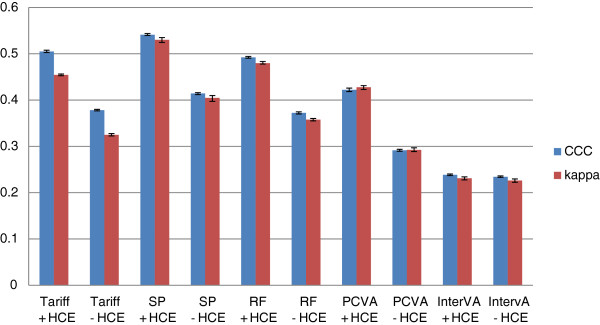
**Histogram of adult Cohen’s kappa and CCC for 5 analytical methods across 500 splits.** Comparative performance of five methods according to Cohen’s kappa (%) and chance-corrected concordance (%) for adult causes with and without health care experience (HCE).

Figure 5 provides Cohen’s Kappa and CCC for each method (except King-Lu) which gives an overall summary of the performance of each method in assigning individual deaths to their true cause. CCC and kappa are reported both in cases where household recall of HCE is available and not. Overall, SSP does the best at assigning individual causes of death with or without HCE. Tariff and RF have slightly lower performance followed by PCVA, and InterVA-4 is substantially worse. These general orderings of the methods are similar whether performance is assessed with or without HCE using kappa or CCC as the metric.

Additional file 9: Table S4 shows the relationship between the estimated and true CSMFs across 500 test-train splits for adults. The relationships are quantified in terms of the slope, intercept, RMSE of the regression of estimated CSMF on true CSMF and the average absolute error in CSMFs across all splits. There are some general findings that hold across all methods, including the observation that other non-communicable diseases have among the largest absolute errors as compared to other causes while injuries such as road traffic accidents have the lowest absolute error. Simple inspection confirms that RF with HCE has the smallest absolute error for 9 of the 34 causes. KL, SSP, Tariff, PCVA and InterVA-4 with HCE have the smallest error for five, five, two, two and zero causes, respectively. Additionally, RF ties for smallest absolute error eight times while KL, SSP, Tariff, PCVA and InterVA-4 with HCE tie three, one, three, zero and zero times respectively. Both with and without HCE, InterVA-4 gives large errors for pneumonia and other cardiovascular diseases. Table 1 provides median CSMF accuracy and CCC for each method with uncertainty intervals.

**Table 1:** Median chance-corrected concordance (%), cause-specific mortality fraction accuracy for 6 methods across 500 splits by age and health care experience

		**Tariff**				**SSP**			
		**CCC**		**CSMF accuracy**		**CCC**		**CSMF accuracy**	
		**Median**	**95% CI**	**Median**	**95% CI**	**Median**	**95% CI**	**Median**	**95% CI**
Adult	No HCE	37.8	(37.6, 37.9)	0.717	(0.711, 0.721)	41.4	(41.1, 41.6)	0.715	(0.709, 0.720)
	HCE	50.5	(50.2, 50.7)	0.77	(0.766, 0.775)	54.1	(53.9, 54.3)	0.764	(0.760, 0.769)
Child	No HCE	44.6	(44.2, 45.0)	0.744	(0.736, 0.751)	44.9	(44.5, 45.2)	0.74	(0.735, 0.746)
	HCE	52.5	(52.1, 53.0)	0.783	(0.776, 0.786)	52.1	(51.7, 52.4)	0.768	(0.762, 0.774)
Neonate	No HCE	44.6	(44.2, 44.9)	0.809	(0.801, 0.817)	48.2	(47.9, 48.6)	0.778	(0.768, 0.787)
	HCE	47.3	(46.9, 47.7)	0.817	(0.809, 0.824)	50.3	(50.1, 50.7)	0.79	(0.781, 0.801)
**Cont’d**									
		**RF**				**PCVA**			
		**CCC**		**CSMF accuracy**		**CCC**		**CSMF accuracy**	
		**Median**	**95% CI**	**Median**	**95% CI**	**Median**	**95% CI**	**Median**	**95% CI**
Adult	No HCE	37.2	(37.0, 37.4)	0.708	(0.705, 0.712)	29.1	(28.9, 29.3)	0.638	(0.632, 0.644)
	HCE	49.2	(49.0, 49.4)	0.769	(0.766, 0.774)	42.2	(41.8, 42.5)	0.68	(0.673, 0.687)
Child	No HCE	44.6	(44.1, 44.9)	0.715	(0.706, 0.721)	33.5	(33.2, 33.7)	0.63	(0.615, 0.637)
	HCE	50.2	(49.7, 50.5)	0.739	(0.729, 0.748)	44.5	(44.1, 45.2)	0.678	(0.671, 0.685)
Neonate	No HCE	47.2	(46.9, 47.5)	0.769	(0.759, 0.779)	25.5	(25.2, 26.0)	0.692	(0.682, 0.701)
	HCE	50	(49.8, 50.4)	0.793	(0.779, 0.802)	29.3	(28.7, 29.7)	0.719	(0.707, 0.734)
**Cont’d**									
		**KL**				**InterVA**			
		**CCC**		**CSMF accuracy**		**CCC**		**CSMF accuracy**	
		**Median**	**95% CI**	**Median**	**95% CI**	**Median**	**95% CI**	**Median**	**95% CI**
Adult	No HCE			0.672	(0.667, 0.676)	23.4	(23.3, 23.6)	0.611	(0.605, 0.620)
	HCE			0.688	(0.682, 0.692)	23.8	(23.6, 24.0)	0.625	(0.617, 0.632)
Child	No HCE			0.674	(0.668, 0.680)	29.6	(29.3, 29.9)	0.514	(0.506, 0.526)
	HCE			0.69	(0.683, 0.696)	30.3	(30.0, 30.6)	0.52	(0.510, 0.529)
Neonate	No HCE			0.808	(0.796, 0.817)	20	(19.8, 20.4)	0.627	(0.602, 0.641)
	HCE			0.81	(0.799, 0.819)	19.4	(19.2, 19.8)	0.629	(0.606, 0.648)
King-Lu (KL) does not estimate individual causes so chance-corrected concordance and Cohen's kappa cannot be calculated.									

Table 1 shows chance-corrected concordance (CCC), and cause-specific mortality fraction (CSMF) accuracy for all methods across 500 splits by age and health care experience for adults, children, and neonates. CI, confidence interval; KL, King-Lu; PCVA, physician-certified VA; RF, Random Forest; SSP, Simplified Symptom Pattern; VA, verbal autopsy.

**Figure 6:**
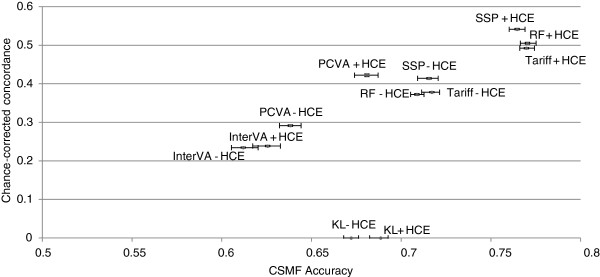
**Plot of adult CCC and CSMF accuracy for 6 analytical methods across 500 splits.** Comparative performance of six methods according to chance-corrected concordance (%) and cause-specific mortality fraction accuracy for adult causes with and without health care experience (HCE). CCC, chance-corrected concordance; CSMF, cause-specific mortality fraction.

Figure 6 compares the performance of the six VA methods for adult cause of death assignment in terms of performance in assigning cause of death for individual deaths (CCC) and performance in estimating CSMFs (CSMF accuracy). Across the VA analytical methods, there is marked variation in both CCC and CSMF accuracy. In general, these two performance dimensions are highly correlated. Although RF performs best for CSMF accuracy in adults, Tariff performs nearly as well (a difference of 0.008), a margin that is well within the 95% uncertainty interval for the median across the 500 splits for each method. SSP performs best for CCC, followed by RF. These three methods, with the inclusion of HCE, outperform all other methods for both CCC and CSMF accuracy. Even without HCE, Tariff, RF and SSP perform better than PCVA, King-Lu and InterVA-4 in terms of CSMF accuracy, and they perform better than InterVA-4 in terms of CCC. In fact, InterVA-4 performs notably poorly for both CCC and CSMF accuracy compared to all other methods, including PCVA.

### Children

**Figure 7:**
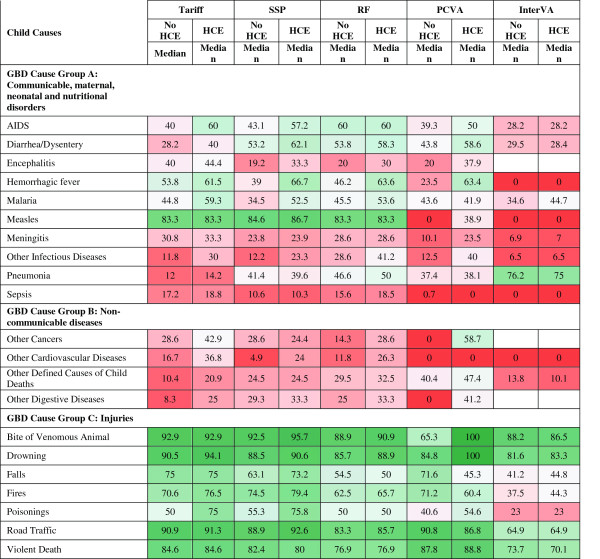
**Sensitivity (%) for 5 methods for 21 child causes.** Figure 7 shows the median sensitivity for each method (except King-Lu which does not provide individual cause of death assignments) for 21 child causes. Cells are shaded from red (low sensitivity) to green (high sensitivity) to help identify the relative differences between sensitivities across methods and causes. GBD, Global Burden of Disease; HCE, health care experience; PCVA, physician-certified VA; RF, Random Forest; SSP, Simplified Symptom Pattern; VA, verbal autopsy.

**Figure 8:**
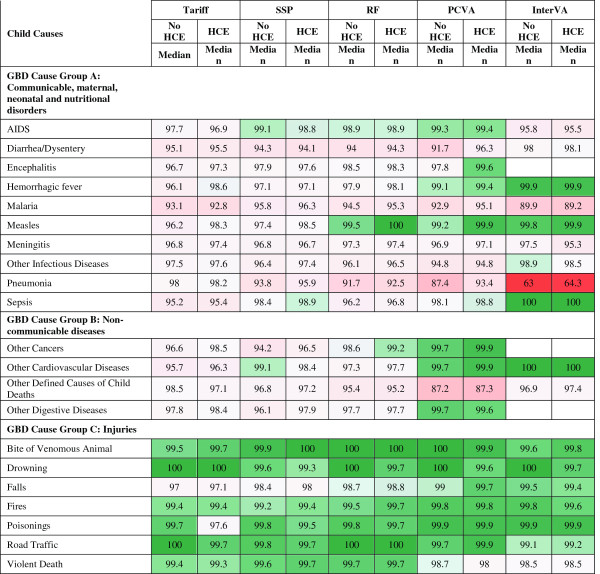
**Specificity (%) for 5 methods for 21 child causes.** Figure 8 shows the median specificity for each method (except King-Lu which does not provide individual cause of death assignments) for 21 child causes. Cells are shaded from red (low specificity) to green (high specificity) to help identify the relative differences between specificities across methods and causes. GBD, Global Burden of Disease; HCE, health care experience; PCVA, physician-certified VA; RF, Random Forest; SSP, Simplified Symptom Pattern; VA, verbal autopsy.

**Figure 9:**
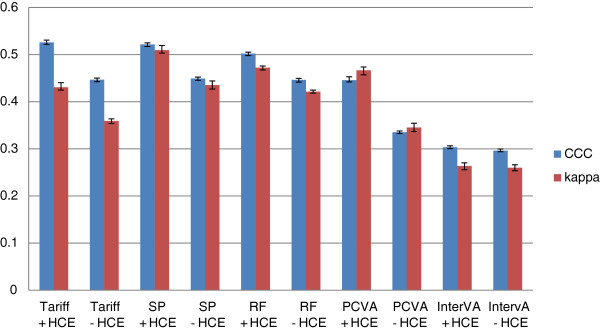
**Histogram of child Cohen’s kappa and CCC for 5 analytical methods across 500 splits.** Comparative performance of five methods according to Cohen’s kappa (%) and chance-corrected concordance (%) for child causes with and without health care experience (HCE). CCC, chance-corrected concordance.

Figure 7 provides the sensitivities by cause for child deaths for the five methods that assign cause at the individual level. For 12 causes, SSP with HCE yields sensitivities greater than 50%, including bites of venomous animals, road traffic injuries, measles, drowning, violent death, falls, fires, poisonings, hemorrhagic fever, diarrhea, AIDS and malaria. Interestingly, pneumonia has sensitivities ranging from 14.2% for Tariff with HCE to 75.0% for InterVA-4 with HCE across methods. Although it performs comparatively poorly overall, InterVA-4 has substantially higher sensitivities for pneumonia than other methods, with and without HCE; however, this must be viewed with respect to the extremely low specificity of InterVA-4 for pneumonia which leads to a very large fraction of deaths to pneumonia regardless of the true pneumonia CSMF, as shown in Figure 8. The method yielding the highest sensitivity for each cause varies: SSP is the best for six causes, Tariff is best for six causes, PCVA is the best for six, InterVA-4 is best for one, RF is the best for one, and RF and Tariff are tied for one. Figure 9 summarizes the overall performance of each of the methods at assigning individual causes of death using kappa and CCC. Depending on the metric, Tariff or SSP appears to have the best performance; the differences across methods appear less consistent than for adults with the exception that InterVA-4 has substantially poorer performance than the other four methods.

Additional file 10: Table S5 provides, the intercept, slope and RMSE of a linear regression between the estimated CSMF and true CSMF as well as the average absolute error between true and estimated CSMF, across the various methods. For 4 of 21 causes, RF has the smallest errors, and Tariff has the smallest errors for seven of them. There is marked variation across methods for some important childhood causes. For example, for diarrhea, Tariff has much smaller errors, especially when compared to SSP and InterVA-4. For pneumonia, SSP does much better than the other methods; notably, InterVA-4 does very poorly with an average absolute error of 33.0 percentage points. This suggests that the high sensitivity for InterVA-4 for pneumonia arises because the method tends to over assign many child deaths to pneumonia. This is corroborated by the comparatively lower specificity for this cause and method as seen in Figure 8. For malaria, KL does relatively well, and Tariff and InterVA-4 have larger errors.

**Figure 10:**
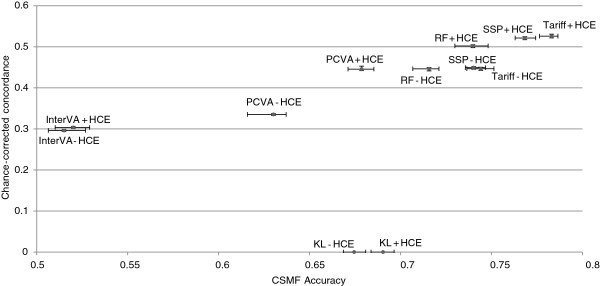
**Plot of child CCC and CSMF accuracy for 6 analytical methods across 500 splits.** Comparative performance of six methods according to chance-corrected concordance (%) and cause-specific mortality fraction accuracy for child causes with and without health care experience (HCE). CCC, chance-corrected concordance; CSMF, cause-specific mortality fraction.

Overall performance of the different methods for assigning child deaths by cause is summarized in Figure 10 which compares CCC and CSMF accuracy for each of the six methods. The correlation between method performance at the individual level and at the population level for all methods, excluding King-Lu, which does not provide individual cause assignment, is strong (correlation coefficient of 0.954 for adults, 0.952 for children). The highest CCC is achieved by Tariff followed very closely by SSP and RF, while Tariff achieved the highest CSMF accuracy, followed very closely by SSP. InterVA-4 performs markedly worse on both dimensions of VA performance, similarly to what was seen in adults. Likewise, the KL method does substantially worse than Tariff, RF, and SSP with or without HCE recall. PCVA for children with or without HCE does much better than InterVA-4 but worse than the other automated methods for CSMF accuracy. For PCVA with HCE, however, the gap in terms of CCC with the better methods is smaller than for CSMF accuracy.

### Neonates and stillbirths

**Figure 11:**
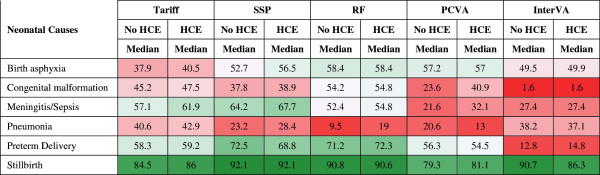
**Sensitivity (%) for five methods for six neonatal causes.** Figure 11 shows the median sensitivity for each method (except King-Lu which does not provide individual cause of death assignments) for five neonatal causes and stillbirth. Cells are shaded from red (low sensitivity) to green (high sensitivity) to help identify the relative differences between sensitivities across methods and causes. HCE, health care experience; PCVA, physician-certified VA; RF, Random Forest; SSP, Simplified Symptom Pattern; VA, verbal autopsy.

**Figure 12:**
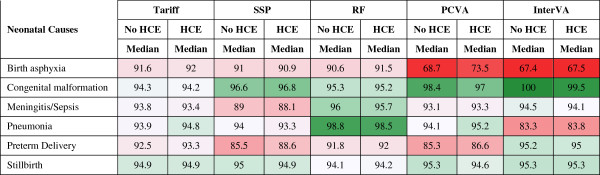
**Specificity (%) for five methods for six neonatal causes.** Figure 12 shows the median specificity for each method (except King-Lu which does not provide individual cause of death assignments) for five neonatal causes and stillbirth. Cells are shaded from red (low specificity) to green (high specificity) to help identify the relative differences between specificities across methods and causes. HCE, health care experience; PCVA, physician-certified VA; RF, Random Forest; SSP, Simplified Symptom Pattern; VA, verbal autopsy.

**Figure 13:**
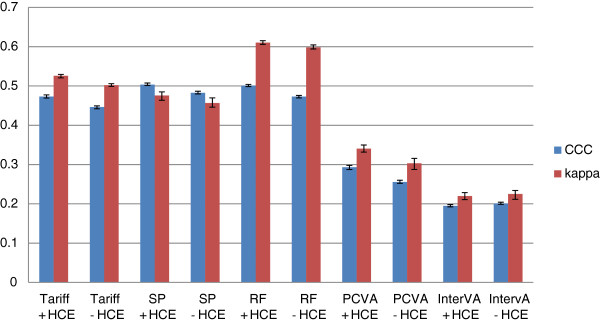
**Histogram of neonate Cohen’s kappa and CCC for 5 analytical methods across 500 splits.** Comparative performance of five methods according to Cohen’s kappa (%) and chance-corrected concordance (%) for neonatal causes with and without health care experience (HCE). CCC, chance-corrected concordance.

**Figure 14:**
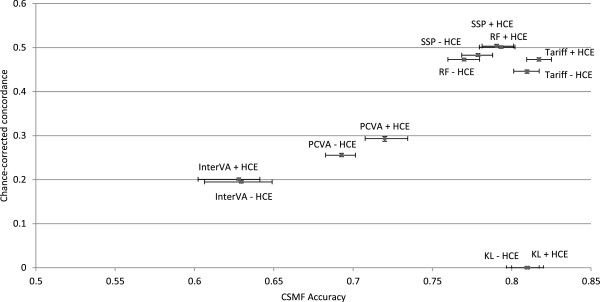
**Plot of neonate CCC and CSMF accuracy for 6 analytical methods across 500 splits.** Comparative performance of six methods according to chance-corrected concordance (%) and cause-specific mortality fraction accuracy for neonate causes with and without health care experience (HCE). CCC, chance-corrected concordance; CSMF, cause-specific mortality fraction.

The neonatal cause list is much shorter, five causes plus stillbirths, compared to the child and adult cause lists. Figures 11 and 12 provide sensitivities and specificities by method. Most methods do well (more than 80% sensitivity) for stillbirths but do particularly poorly in terms of sensitivity for pneumonia, with the Tariff method providing the highest sensitivity of 42.9% with HCE. PCVA and InterVA-4 do not have the highest sensitivity for any cause, regardless of inclusion of HCE. There is marked variation across the methods for performance on congenital anomalies and preterm delivery. In Additional file 11: Table S6, which displays the CSMF regression information for each cause as well as the average absolute error between estimated and true CSMFs across all 500 splits, only Tariff has average absolute errors below 0.05 across all causes. Figure 13 compares the performance of the five methods in terms of CCC and Cohen’s kappa, and Figure 14 compares for neonates the overall performance across causes in terms of CCC and CSMF accuracy. Tariff yields the highest level of CSMF accuracy, followed by KL, although the difference between them is not statistically significant. SSP achieves the highest CCC, outperforming RF by a small margin.

### Performance of methods across different underlying cause compositions

Table 2 shows how many times each method performs best across the 500 test-train splits for adults, children and neonates, with and without HCE, both for CCC and for CSMF accuracy. Because all methods may do better or worse for particular CSMF compositions in the test dataset, this provides an assessment that indicates how often across a wide range of CSMF compositions each method performs best. For CCC in adults, SSP performs best for more than 99.0% of splits. In children, Tariff does better than SSP and RF. In neonates, SSP performs best, followed by RF. In no case does InterVA-4 provide the highest overall CCC and in only 5.4% of cases of child death with HCE does PCVA provide the highest CCC.

**Table 2:** Head-to-head performance of 6 analytical models across 500 splits (number)

		**CSMF accuracy**						**Chance-corrected concordance**				
		**Tariff**	**SSP**	**RF**	**PCVA**	**King-Lu**	**InterVA**	**Tariff**	**SSP**	**RF**	**PCVA**	**InterVA**
**Adult**	**No HCE**	162	156	150	8	13	11	5	493	2	0	0
	**HCE**	156	142	189	10	0	3	4	495	1	0	0
**Child**	**No HCE**	232	166	68	15	18	1	169	195	136	0	0
	**HCE**	264	141	69	21	5	0	236	191	55	18	0
**Neonate**	**No HCE**	203	44	44	24	163	22	46	300	154	0	0
	**HCE**	201	50	62	30	138	19	47	254	199	0	0

Table 2 gives the number of 500 test-train datasets for which each method performs best for chance-corrected concordance (CCC) and cause-specific mortality fraction (CSMF) accuracy, by age and health care experience (HCE). King-Lu (KL) does not estimate individual causes so chance-corrected concordance cannot be calculated. PCVA, physician-certified VA; RF, Random Forest; SSP, Simplified Symptom Pattern; VA, verbal autopsy.

In terms of CSMF accuracy, taking into account variation in the cause composition leads to quite different results for adults than for children and neonates. Among adults, RF performs best 30.0% of the time without HCE and 37.8% of the time with HCE. Tariff does best 32.3% of the time with HCE and 32.4% of the time without HCE, and SSP in 28.4% of cases with HCE and 31.2% without HCE. For children, Tariff has the highest CSMF accuracy 52.8% of the time with HCE, SSP is the highest just under 28.3% of the time, and RF is the highest in 13.8% of the draws. The advantage of Tariff over other methods is more pronounced in neonates, where it has the highest CSMF accuracy in 40.2% or more of the cases with HCE, while King-Lu provides the highest CSMF accuracy 27.6% of the time.

For adults, children and neonates, the findings of this analysis across different cause compositions closely aligned with the results of the comparative performance of the six different methods examining only the median performance. Overall, in 6,000 head-to-head comparisons across the three age groups, with and without HCE, for CCC and for CSMF accuracy, SSP performed best in 43.8%, Tariff performed best in 28.8%, RF in 18.8% of the tests, PCVA in 2.1%, King-Lu in 5.6%, and InterVA-4 in 0.9%. These figures, however, tend to mask the fact that SSP does very well on CCC in adults, while RF does well on CSMF accuracy. Tariff does well on CCC in children with HCE, and CSMF accuracy in children and neonates with and without HCE. SSP does well in CCC for neonates with and without HCE. Overall, SSP does the best for CCC, performing best in 1,928 of the 3,000 comparisons, and Tariff does best for CSMF accuracy, performing best in 1,218 of the 3,000 comparisons for CSMF accuracy.

Additional files 12, 13 and 14: Tables S7 to S9 contain a similar comparison of minimum absolute errors by cause of death. These tables show how many times each analytic method produces the smallest absolute error between the true and estimated CSMF for each cause. In the case of a tie for smallest absolute error for a given split, we assigned a portion of the ‘credit’ for that split to each method, resulting in non-integer number values for some methods. SSP produces the highest number of smallest absolute errors for adult causes of death for analyses of VAs with and without health care experience in 22.5% and 21.6% of the 17,000 comparisons, respectively. For children, the Tariff method does best, with the smallest absolute error in 22.4% of the 10,500 comparisons with HCE and 23.1% of the comparisons without HCE. For neonates, the King-Lu method does best, minimizing the error in 23.1% of the 6,000 comparisons with HCE and 23.6% of the time without HCE.

## Discussion

Our findings that physicians are less accurate than computers in correctly certifying causes of death in the low and middle income populations that we studied are likely to be counter-intuitive. Physicians are specifically trained to understand and recognize pathological processes and, in principle at least, to correctly apply the rules and procedures of the ICD in order to certify the cause of death. Yet, with the single exception of one automated method (Inter-VA-4), we find that physicians are significantly poorer at diagnosing the cause of death from information reported by the household in a VA interview than computer algorithms processing the same information. Why is this, can we be confident in our findings, and what are their implications for monitoring causes of death in populations and measuring progress with development goals?

With rising interest in the use of VA as a tool to monitor causes of death, a range of new analytical methods have become available that offer an alternative to costly and inefficient PCVA and yet perform better. The PHMRC GS VA validation study provides a unique opportunity to quantify and compare the performance of this diverse array of VA analytical methods using a large multisite set of deaths where the cause of death, according to strict clinical and diagnostic criteria, has been reliably established. Methods vary in their performance by cause and age group. However, three methods, Tariff, RF and SSP consistently and significantly provide better CCC and CSMF accuracy than PCVA.

Most published studies and national data collection efforts [25-39] use PCVA. PCVA can be expensive, difficult to organize in settings with few physicians and can take scarce physician resources away from other clinical responsibilities. For example, VA data collected in India from 2001 to 2003 as part of the Sample Registration System was not published until 2010 [40,41] because of the delays in obtaining physician reading of VAs. We show here that PCVA performs worse overall on both CCC and CSMF accuracy than three automated approaches (Tariff, SSP, and RF) for all three age groups with and without HCE. Given that the automated methods are essentially free to apply, can be implemented with effectively no delay and are now increasingly available on a wide set of computational platforms, there would seem to be little scientific, financial or moral justification to continue with PCVA.

This study reports worse performance of PCVA compared to prior studies that have compared PCVA to hospital diagnosis or, frequently, to poor-quality medical records [42-44]. Often hospital diagnosis in resource-poor settings may be based on limited medical imaging, laboratory, or pathological evidence. In fact, the PHMRC study found that even in well-equipped hospitals, only a small percentage of in-hospital deaths met strict clinical and diagnostic criteria. We, therefore, have greater confidence in the diagnostic accuracy of our GS reference cases than criteria used in other studies. In addition, this study uses much more robust and comparable metrics of performance compared to previous studies. For some causes, notably some adult non-communicable diseases, child pneumonia, malaria and neonatal birth asphyxia, PCVA appears to be systematically biased upwards in suggesting larger cause fractions than are present in the population, especially at low true CSMFs.

Our findings suggest that the optimal VA method may depend on the purpose of a particular study. Specific research studies with a strong interest in reliably diagnosing particular causes of death may want to factor in the comparative performance of methods for specific causes, as demonstrated in the tables and figures on sensitivity and average absolute errors. For more general use in cause of death surveillance, however, we believe that the choice of method should place greater emphasis on the ease with which it can be explained to implementers and users. Tariff is likely to be easier for medical practitioners and other users to understand since it is predicated on a common clinical knowledge about the symptoms for each disease. Moreover, specific tariff scores for each cause can be directly examined for plausibility. Tariff can, in principle, be implemented in a spreadsheet so that the logic and approach can be followed more easily than RF and SSP, which require complex machine learning and statistical methods. These communication and training advantages, combined with the best overall performance at the population level, suggest that of the currently available automated methods, Tariff is our preferred method of choice for population health monitoring.

Two automated methods that have been proposed and applied to VA data, InterVA-4 and King-Lu, performed less well than might have been expected. Flaxman *et al*. [16] provide an explanation for the poor performance of King-Lu for adults and children. The King-Lu method does not perform well when more than ten causes are included in the cause list. For InterVA-4, the results of this evaluation are particularly poor, with the method performing best in only 56 out of the 3,000 comparisons for CSMF accuracy and never performing best for comparisons using CCC. Given that both the SSP method and InterVA are constructed from an application of Bayes’ Theorem, why is their performance so different? Lozano *et al*. [15] suggest four reasons: InterVA assumes that all signs and symptoms conditional on the true cause are independent of each other; it uses a restricted set of signs and symptoms compared to the full WHO or PHMRC VA instrument; the probabilities of a given sign or symptom conditional on the true cause are generated from expert opinion rather than data; and it estimates a posterior distribution across all causes at once rather than posterior distributions assessing each cause one at a time against all other causes. We have shown separately that by imposing these restrictive assumptions on the symptom pattern approach, its performance also drops to the level of InterVA [15]. Further, published ‘validation’ studies of InterVA have been comparisons with PCVA and not to a reference or GS as we have used in this assessment. Thus, while InterVA represented an important advance in the use of automated diagnostic approaches for VA, newer empirical approaches now perform dramatically better.

Even using the best performing methods, VA does not perform as well in adults as medical certification of causes of death in a sophisticated hospital. Hernandez *et al*. reported median CCC of 66.5% and a CSMF accuracy of 0.822 in large tertiary hospitals in Mexico [5]. While it is to be expected that the cause of death for hospitals with good diagnostic capacity are likely to be more accurate than VA, the gap in performance is not as large as one might have expected. Causes of death assigned in less sophisticated hospitals might in fact be less accurate than those assigned by RF, SSP or Tariff based on a VA. Even in these tertiary Mexican hospitals, these three methods actually did better than medical certification of causes of death for children and neonates. This suggests that there may even be a role for a structured VA to formally supplement hospital diagnostic information in some settings. In some high-income countries, structured interviews that resemble VA have been used in maternal death audits and the US national mortality follow-back survey [45,46].

The strength of this large, comparative study of the performance of various diagnostic methods, including physician certification applied to VA information is that, for the first time, we can confidently and objectively conclude which methods and measurement approaches perform best in different age groups. These are novel findings of potentially substantial importance for country health monitoring strategies. Nonetheless, there are some potentially important caveats to the comparative assessments reported here. While the PHMRC GS dataset is the largest study of its kind to date and has applied much stricter criteria for cause of death assignment than has been done previously, it was conducted in a limited number of sites in the developing world: Andhra Pradesh and Uttar Pradesh in India, Bohol in the Philippines, Mexico City in Mexico, and Dar es Salaam and Pemba Island in Tanzania. An important potential limitation of this study is that there may be cultural variation in how household members respond to different items in a VA interview. In this study, largely due to sample size, we have not been able to assess validity of different methods for assigning cause of death by specific site. The real possibility of cultural variation means that we must be careful in generalizing results on VA method performance observed in these six sites to all other populations where VA might be used. Further research that collects more deaths with cause of death assignment following strict clinical and diagnostic criteria in other sites would strengthen the generalizability of these findings. Nevertheless, the higher performing VA methods, such as RF, SSP and the easily understood Tariff method, appear to have consistently performed better than other options. A further limitation is that only deaths with extensive documentation to meet the GS diagnostic criteria were included; in most cases these deaths occurred in a hospital. Household members may respond to VA questions differently if the death occurs without any medical care; the signs and symptoms of individuals who tend to go to a hospital for care may be different, or reported differently, than for deaths outside hospitals from the same cause of death. Both of these limitations, however, apply to all VA validation studies. In this comparative assessment, removing any information about HCE from the assessment could be viewed as a proxy for the performance of VA methods for deaths without contact with health services although there still remains the possibility that HCE may change responses to the structured part of the VA. Even so, removing information on HCE did not change the ordering of the methods in most cases.

Given that these automated methods are operationally easier and less costly to implement than PCVA and have demonstrably better performance, we believe that the time has come for their broader application in routine health information systems as well as in field research. Indeed, as automated methods continue to evolve and become simpler to implement, the operational barriers to their application will become progressively less important. Two factors will aid this greater dissemination and use by countries: strategic dissemination about successful application of the current methods by countries where they are needed and, perhaps more importantly, progress toward simplifying data collection instruments using criteria that preserve performance but significantly reduce interview time. Initial results from item reduction approaches suggest that the current PHMRC interview instrument could be reduced by about two-fifths without any significant loss of performance. Further research is urgently needed to determine how questionnaires can be further reduced and at what cost in terms of performance. The PHMRC dataset can be used to aid in some of this item reduction research. Another important area for improvement is to simplify the collection of the open text information in the VA instrument. For example, words with high tariffs that are identified in the open text could in many cases be converted to structured items. Ideally, the open text component could be dropped facilitating data collection and digital transcription if enough of the information content used by the automated methods could be converted into structured items.

The findings presented here, particularly on the three top performing methods Tariff, SSP, and RF, suggest a range of ways these results could be used to improve cause of death estimation through further research. As in other analytical applications and fields [47], blends or ensembles of these approaches may in fact perform better [48,49]. In an automated environment, implementing VA ensembles will be relatively simple and further research on this should be a high priority. Another area of investigation is the systematic correction of estimated cause fractions from a method using the known biases from the methods. Additional files 9, 10, and 11: Tables S4 to S6 provide detail on the relationship across the 500 test datasets between the estimated CSMFs and true CSMFs from each method for adults, children and neonates. This type of information could be used to back-correct CSMFs. Such back correction would, on average, improve the accuracy of estimated CSMFs but in some cases would make them less accurate. The PHMRC dataset, which is available in the public domain, should stimulate further methods innovation.

## Conclusions

Drawing on the largest, most culturally diverse validation data set of neonatal, child and adult deaths ever assembled in developing countries, for which the underlying cause of death had been reliably established using standardized and strict clinical criteria, we have shown that automated methods, not involving physician judgment, significantly outperform physicians and commonly used methods such as Inter-VA in correctly diagnosing the cause of death. The methods allow rapid, standardized, efficient and comparable cause of death data to be generated for populations where the vast majority of deaths occur with limited medical attention. One of these methods in particular, the Tariff method, is well suited for widespread application in routine mortality surveillance systems given its simplicity and consistent high performance, as assessed by strict statistical criteria.

The past five years have seen a rapid expansion of alternative approaches to VA. We should expect and encourage this innovation. Undoubtedly, future methodological research would benefit from an expanded GS database of cases drawn from different populations and for different causes than those collected for the PHMRC study. These developments, along with improved operational methods for data collection, will greatly facilitate the widespread adoption of VA by countries in which there is currently vast ignorance regarding cause of death patterns and how these patterns are changing. We see this as a fundamental component of the ‘data revolution’ that is much discussed, and propagated, as a key requirement for assuring accountability in the post-2015 development agenda. Indeed, knowledge about causes of death in less developed populations could be rapidly and vastly improved through the immediate application of the comparatively cost-effective, standardized, automated, and validated methods reported here.

## Competing interests

The authors declare that they have no conflicts of interest.

## Authors’ contributions

CJLM, RB and ADL designed the overall PHMRC study. CJLM, ADL, RB, SMA, AB, LD, ED, GD, VD, UD, AD, WF, ADF, SG, BH, RJ, HDK, AK, VK, ML, RL, SM, BN, SLO, DP, ZP, DRV, HR, IR, MR, MS, DS, SS and VT were part of the PHMRC and contributed to study design and/or fieldwork to collect the gold standard dataset and verbal autopsy interviews. CJLM, RL, ADL, ADF, BH, SLJ, PS, DP and AS worked on the comparison of methods. Improvements to the Tariff method were designed by PS. The first draft of the paper was written by CJLM, ADL and RL. CA contributed to the analysis, produced tables and figures, referenced the final draft and managed the data collected through the PHMRC. AV and MKF contributed to the analysis and provided conceptual feedback. SLO contributed to the drafts, managed the analysis and coordinated the PHMRC study. All authors reviewed the draft and provided comments. All authors read and approved the final manuscript.

## Supplementary Material

Additional file 1: Table S1a Number of adult gold standard deaths collected in the PHMRC study by diagnostic level and cause. Click here for file

Additional file 2: Table S1b Number of child gold standard deaths collected in PHMRC study by diagnostic level and cause. Click here for file

Additional file 3: Table S1c Number of neonate gold standard deaths collected in PHMRC study by diagnostic level and cause. Click here for file

Additional file 4: Table S2a Map of InterVA-4 adult cause list to PHMRC adult cause list. Click here for file

Additional file 5: Table S2b Map of InterVA-4 child cause list to PHMRC child cause list. Click here for file

Additional file 6: Table S2c Map of InterVA-4 neonate cause list to PHMRC neonate cause list. Click here for file

Additional file 7 **Additional Document 1.** Updates in the Tariff Method since publication of the PHMRC paper. Click here for file

Additional file 8: Table S3 Uncertainty intervals (UI) and standard deviations (SD) around estimates of sensitivity and specificity across 500 splits for adult, child and neonate VAs. Click here for file

Additional file 9: Table S4 Comparison of estimated versus true adult population cause fractions by method. Click here for file

Additional file 10: Table S5 Comparison of estimated versus true child population cause fractions by method. Click here for file

Additional file 11: Table S6 Comparison of estimated versus true neonatal population cause fractions by method. Click here for file

Additional file 12: Table S7 Head-to-head performance of 6 analytical models for adults across 500 splits using mean absolute error (number). Click here for file

Additional file 13: Table S8 Head-to-head performance of 6 analytical models for children across 500 splits using mean absolute error (number). Click here for file

Additional file 14: Table S9 Head-to-head performance of 6 analytical models for neonates across 500 splits using mean absolute error (number). Click here for file

## References

[B1] Ruzicka LT Lopez AD The use of cause-of-death statistics for health situation assessment: national and international experiences World Health Stat Q 1990 43 249 258 2293493

[B2] Mathers CD Fat DM Inoue M Rao C Lopez AD Counting the dead and what they died from: an assessment of the global status of cause of death data Bull World Health Organ 2005 83 171 177 15798840 PMC2624200

[B3] Mahapatra P Shibuya K Lopez AD Coullare F Notzon FC Rao C Szreter S Civil registration systems and vital statistics: successes and missed opportunities Lancet 2007 370 1653 1663 10.1016/S0140-6736(07)61308-7 18029006

[B4] United Nations A New Global Partnership: Eradicate Poverty and Transform Economies Through Sustainable Development 2013 New York, NY: United Nations

[B5] Hernández B Ramírez-Villalobos D Romero M Gómez S Atkinson C Lozano R Assessing quality of medical death certification: concordance between gold standard diagnosis and underlying cause of death in selected Mexican hospitals Popul Health Metr 2011 9 38 10.1186/1478-7954-9-38 21816103 PMC3160931

[B6] Google books Ngram viewer [http://books.google.com/ngrams/graph?content=Verbal+autopsy&year_start=1950&year_end=2008&corpus=15&smoothing=5&share=]

[B7] Instituto Nacional de Estatística Mortalidade em Mocambique: Inquerito Nacional sobre Causas de Mortalidade, 2007/8 2009 Mozambique

[B8] Lopez AD Counting the dead in China BMJ 1998 317 1399 1400 10.1136/bmj.317.7170.1399 9822388 PMC1114289

[B9] Yang G Hu J Rao KQ Ma J Rao C Lopez AD Mortality registration and surveillance in China: history, current situation and challenges Popul Health Metr 2005 3 3 10.1186/1478-7954-3-3 15769298 PMC555951

[B10] Baiden F Bawah A Biai S Binka F Boerma T Byass P Chandramohan D Chatterji S Engmann C Greet D Jakob R Kahn K Kunii O Lopez AD Murray CJL Nahlen B Rao C Sankoh O Setel PW Shibuya K Soleman N Wright L Yang G Setting international standards for verbal autopsy Bull World Health Organ 2007 85 570 571 10.2471/BLT.07.043745 17768508 PMC2636382

[B11] World Health Organization Verbal Autopsy Standards: The 2012 WHO Verbal Autopsy Instrument Release Candidate 1 Available at: http://www.who.int/healthinfo/statistics/WHO_VA_2012_RC1_Instrument.pdf

[B12] INDEPTH Verbal Autopsy Instruments [http://www.indepth-network.org/Resource Kit/INDEPTH DSS Resource Kit/INDEPTHVerbalAutopsyInstruments.htm]

[B13] Murray CJ Lopez AD Black R Ahuja R Ali SM Baqui A Dandona L Dantzer E Das V Dhingra U Dutta A Fawzi W Flaxman AD Gómez S Hernández B Joshi R Kalter H Kumar A Kumar V Lozano R Lucero M Mehta S Neal B Ohno SL Prasad R Praveen D Premji Z Ramírez-Villalobos D Remolador H Riley I Population Health Metrics Research Consortium gold standard verbal autopsy validation study: design, implementation, and development of analysis datasets Popul Health Metr 2011 9 27 10.1186/1478-7954-9-27 21816095 PMC3160920

[B14] Lozano R Lopez AD Atkinson C Naghavi M Flaxman AD Murray CJ Performance of physician-certified verbal autopsies: multisite validation study using clinical diagnostic gold standards Popul Health Metr 2011 9 32 10.1186/1478-7954-9-32 21816104 PMC3160925

[B15] Lozano R Freeman MK James SL Campbell B Lopez AD Flaxman AD Murray CJ Performance of InterVA for assigning causes of death to verbal autopsies: multisite validation study using clinical diagnostic gold standards Popul Health Metr 2011 9 50 10.1186/1478-7954-9-50 21819580 PMC3160943

[B16] Flaxman AD Vahdatpour A James SL Birnbaum JK Murray CJ Direct estimation of cause-specific mortality fractions from verbal autopsies: multisite validation study using clinical diagnostic gold standards Popul Health Metr 2011 9 35 10.1186/1478-7954-9-35 21816098 PMC3160928

[B17] James SL Flaxman AD Murray CJ Performance of the Tariff Method: validation of a simple additive algorithm for analysis of verbal autopsies Popul Health Metr 2011 9 31 10.1186/1478-7954-9-31 21816107 PMC3160924

[B18] Flaxman AD Vahdatpour A Green S James SL Murray CJ Random forests for verbal autopsy analysis: multisite validation study using clinical diagnostic gold standards Popul Health Metr 2011 9 29 10.1186/1478-7954-9-29 21816105 PMC3160922

[B19] Murray CJ James SL Birnbaum JK Freeman MK Lozano R Lopez AD Simplified Symptom Pattern Method for verbal autopsy analysis: multisite validation study using clinical diagnostic gold standards Popul Health Metr 2011 9 30 10.1186/1478-7954-9-30 21816099 PMC3160923

[B20] Murray CJ Lozano R Flaxman AD Vahdatpour A Lopez AD Robust metrics for assessing the performance of different verbal autopsy cause assignment methods in validation studies Popul Health Metr 2011 9 28 10.1186/1478-7954-9-28 21816106 PMC3160921

[B21] Byass P Chandramohan D Clark SJ D’Ambruoso L Fottrell E Graham WJ Herbst AJ Hodgson A Hounton S Kahn K Krishnan A Leitao J Odhiambo F Sankoh OA Tollman SM Strengthening standardised interpretation of verbal autopsy data: the new InterVA-4 tool Glob Health Action 2012 5 1 8 22944365 10.3402/gha.v5i0.19281 PMC3433652

[B22] Murray CJ Ezzati M Flaxman AD Lim S Lozano R Michaud C Naghavi M Salomon JA Shibuya K Vos T Wikler D Lopez AD GBD 2010: design, definitions, and metrics Lancet 2012 380 2063 2066 10.1016/S0140-6736(12)61899-6 23245602

[B23] Chandramohan D Setel P Quigley M Effect of misclassification of causes of death in verbal autopsy: can it be adjusted? Int J Epidemiol 2001 30 509 514 10.1093/ije/30.3.509 11416073

[B24] R Development Core Team R: A Language and Environment for Statistical Computing 2010 Vienna, Austria: R Foundation for Statistical Computing

[B25] Setel PW Whiting DR Hemed Y Chandramohan D Wolfson LJ Alberti KGMM Lopez AD Validity of verbal autopsy procedures for determining cause of death in Tanzania Trop Med Int Health 2006 11 681 696 10.1111/j.1365-3156.2006.01603.x 16640621

[B26] Rao C Porapakkham Y Pattaraarchachai J Polprasert W Swampunyalert N Lopez A Verifying causes of death in Thailand: rationale and methods for empirical investigation Popul Health Metr 2010 8 11 10.1186/1478-7954-8-11 20482758 PMC2880956

[B27] Polprasert W Rao C Adair T Pattaraarchachai J Porapakkham Y Lopez A Cause-of-death ascertainment for deaths that occur outside hospitals in Thailand: application of verbal autopsy methods Popu Health Metr 2010 8 13 10.1186/1478-7954-8-13 PMC2881896 20482760

[B28] Joshi R Cardona M Iyengar S Sukumar A Raju CR Raju KR Raju K Reddy KS Lopez A Neal B Chronic diseases now a leading cause of death in rural India–mortality data from the Andhra Pradesh Rural Health Initiative Int J Epidemiol 2006 35 1522 1529 10.1093/ije/dyl168 16997852

[B29] Gajalakshmi V Peto R Verbal autopsy of 80,000 adult deaths in Tamilnadu, South India BMC Public Health 2004 4 47 10.1186/1471-2458-4-47 15488138 PMC535898

[B30] Gajalakshmi V Peto R Kanaka S Balasubramanian S Verbal autopsy of 48 000 adult deaths attributable to medical causes in Chennai (formerly Madras), India BMC Public Health 2002 2 7 10.1186/1471-2458-2-7 12014994 PMC113750

[B31] Jha P Gajalakshmi V Gupta PC Kumar R Mony P Dhingra N Peto R Prospective study of one million deaths in India: rationale, design, and validation results PLoS Med 2006 3 e18 10.1371/journal.pmed.0030018 16354108 PMC1316066

[B32] Ngo AD Rao C Hoa NP Adair T Chuc NTK Mortality patterns in Vietnam, 2006: findings from a national verbal autopsy survey BMC Res Notes 2010 3 78 10.1186/1756-0500-3-78 20236551 PMC2851717

[B33] Morris SK Bassani DG Awasthi S Kumar R Shet A Suraweera W Jha P Diarrhea, pneumonia, and infectious disease mortality in children aged 5 to 14 years in India PLoS One 2011 6 e20119 10.1371/journal.pone.0020119 21629660 PMC3101242

[B34] Campos D França E Loschi RH de Souza FM [Verbal autopsy for investigating deaths from ill-defined causes in Minas Gerais State, Brazil] Cad Saude Publica 2010 26 1221 1233 10.1590/S0102-311X2010000600015 20657986

[B35] Asuzu MC Johnson OO Owoaje ET Kaufman JS Rotimi C Cooper RS The Idikan adult mortality study Afr J Med Med Sci 2000 29 115 118 11379440

[B36] Kodio B de Bernis L Ba M Ronsmans C Pison G Etard JF Levels and causes of maternal mortality in Senegal Trop Med Int Health 2002 7 499 505 10.1046/j.1365-3156.2002.00892.x 12031071

[B37] Chowdhury HR Thompson S Ali M Alam N Yunus M Streatfield PK Causes of neonatal deaths in a rural subdistrict of Bangladesh: implications for intervention J Health Popul Nutr 2010 28 375 382 20824981 10.3329/jhpn.v28i4.6044 PMC2965329

[B38] Kumar R Kumar D Jagnoor J Aggarwal AK Lakshmi PVM Epidemiological transition in a rural community of northern India: 18-year mortality surveillance using verbal autopsy J Epidemiol Community Health 2011 66 890 893 22052938 10.1136/jech-2011-200336

[B39] Engmann C Garces A Jehan I Ditekemena J Phiri M Mazariegos M Chomba E Pasha O Tshefu A McClure EM Thorsten V Chakraborty H Goldenberg RL Bose C Carlo WA Wright LL Causes of community stillbirths and early neonatal deaths in low-income countries using verbal autopsy: an International, Multicenter Study J Perinatol 2011 32 585 592 22076413 10.1038/jp.2011.154 PMC3922534

[B40] Bassani DG Kumar R Awasthi S Morris SK Paul VK Shet A Ram U Gaffey MF Black RE Jha P Causes of neonatal and child mortality in India: a nationally representative mortality survey Lancet 2010 376 1853 1860 21075444 10.1016/S0140-6736(10)61461-4 PMC3042727

[B41] Dhingra N Jha P Sharma VP Cohen AA Jotkar RM Rodriguez PS Bassani DG Suraweera W Laxminarayan R Peto R Adult and child malaria mortality in India: a nationally representative mortality survey Lancet 2010 376 1768 1774 10.1016/S0140-6736(10)60831-8 20970179 PMC3021416

[B42] Yang G Rao C Ma J Wang L Wan X Dubrovsky G Lopez AD Validation of verbal autopsy procedures for adult deaths in China Int J Epidemiol 2006 35 741 748 10.1093/ije/dyi181 16144861

[B43] Pattaraarchachai J Rao C Polprasert W Porapakkham Y Pao-In W Singwerathum N Lopez AD Cause-specific mortality patterns among hospital deaths in Thailand: validating routine death certification Popul Health Metr 2010 8 12 10.1186/1478-7954-8-12 20482759 PMC2881895

[B44] Khosravi A Rao C Naghavi M Taylor R Jafari N Lopez A Impact of misclassification on measures of cardiovascular disease mortality in the Islamic Republic of Iran: a cross-sectional study Bull World Health Organ 2008 86 688 696 10.2471/BLT.07.046532 18797644 PMC2649498

[B45] Gaskin IM Maternal death in the United States: a problem solved or a problem ignored? J Perinat Educ 2008 17 9 13 10.1624/105812408X298336 19252683 PMC2409165

[B46] NVSS - National Mortality Followback Survey [http://www.cdc.gov/nchs/nvss/nmfs.htm]

[B47] Foreman KJ Lozano R Lopez AD Murray CJ Modeling causes of death: an integrated approach using CODEm Popul Health Metr 2012 10 1 10.1186/1478-7954-10-1 22226226 PMC3315398

[B48] Bell RM Koren Y Lessons from the Netflix prize challenge SIGKDD Explor Newsl 2007 9 75 79 10.1145/1345448.1345465

[B49] Krishnamurti TN Kishtawal CM Zhang Z LaRow T Bachiochi D Williford E Gadgil S Surendran S Multimodel ensemble forecasts for weather and seasonal climate J Climate 2000 13 4196 4216 10.1175/1520-0442(2000)013<4196:MEFFWA>2.0.CO;2

