# Supplementary material for: Using verbal autopsy to measure causes of death: the comparative performance of existing methods
Source: BMC Med. 2014 Jan 9;12:5. doi: 10.1186/1741-7015-12-5 (PMC3891983; doi:10.1186/1741-7015-12-5)
Supplement: Additional file 7 — Additional Document 1. Updates in the Tariff Method since publication of the PHMRC paper. [file 1741-7015-12-5-S7.pdf]

Additional file 7: Document 1. Updates to the Tariff method since publication of the PHMRC study.

The Tariff method was developed to use a simple algorithm trained on a pool of validated verbal autopsy data to identify cause of death using binary symptom variables from the Population Health Metrics Research Consortium's (PHMRC) Verbal Autopsy Instrument (VAI).[1,2] Since the publication of this method in 2011, three minor changes have been implemented.

One such change was the introduction of significance testing for each tariff. Five hundred bootstrapped samples of symptom data were used to recreate the tariff matrix used within the algorithm. If the tariff's 95% confidence interval for a particular symptom and cause overlapped with zero, that tariff was defined insignificant and dropped from the algorithm.

Additionally, constraints were added to disallow biologically impossible cause of death assignments. These logical constraints included disallowing males from being assigned to breast cancer, cervical cancer, anemia, hemorrhage, hypertensive disease, other pregnancy-related, or sepsis; disallowing females from being assigned to prostate cancer; and disallowing neonates from being assigned to still birth if aged one day or over.

Finally, flexibility was added to allow for classification of indeterminate cause of death. Originally, the ranking procedure that was laid out by James et.al., involved comparing the cause-specific tariff score of a test observation as compared to cause-specific tariff scores of the training data set that had been resampled with replacement to create a uniform cause distribution. Since publication, cause-specific cutoffs have been imposed to define cases in which no rank was high enough to indicate reasonable confidence in the model selection. If a decedent's rank for a particular cause was not at least as low as the cutoff within a training dataset with uniform cause distributions, then the decedent was disallowed from being assigned to that cause. Further, if no cause received a rank which was at least as low as the specified cutoff, the decedent's cause of death was classified as indeterminate. These cutoffs were percentiles based on the ranks within the uniform training set: the 89th percentile in adults, 95th percentile for children, and the 91st percentile in neonates. Less stringent cutoffs were used when comparing the rank of the decedent by cause to the ranks in the train data set overall (regardless of cause): the 18th percentile in adults, 17th percentile for children, and the 35th percentile in neonates. Sensitivity analyses were used to determine these cutoffs by replicating results for all possible cause-specific and overall cutoff combinations and using an indifference function to optimize the validity metrics of chance-corrected concordance and CSMF accuracy along with the fraction of the test population assigned to indeterminate. By placing cause-specific and overall cutoffs within the ranking procedure, the algorithm was protected from assigning a cause of death based on weak signal from the data.

References

1 James SL, Flaxman AD, Murray CJ. Performance of the Tariff Method: validation of a simple additive algorithm for analysis of verbal autopsies. *Population Health Metrics* 2011; **9**: 31.

2 Murray CJ, Lopez AD, Black R, *et al.* Population Health Metrics Research Consortium gold standard verbal autopsy validation study: design, implementation, and development of analysis datasets. *Population Health Metrics* 2011; **9**: 27.
